# 3-Phenyl-2-thioxo-1,3-thia­zolidin-4-one

**DOI:** 10.1107/S1600536808030079

**Published:** 2008-09-24

**Authors:** Feng-Xia Zhu, Jian-Feng Zhou, Gui-Xia Gong

**Affiliations:** a Jiangsu Key Laboratory for the Chemistry of Low-dimensional Materials, Department of Chemistry, Huaiyin Teachers College, 111 West Changjiang Road, Huaian 223300, Jiangsu, People’s Republic of China; b Key Laboratory of Organic Synthesis of Jiangsu Province, College of Chemistry and Chemical Engineering, Suzhou 215123, People’s Republic of China

## Abstract

In the mol­ecule of the title compound, C_9_H_7_NOS_2_, the heterocycle and the phenyl ring are oriented at a dihedral angle of 72.3 (1)°. Adjacent mol­ecules are connected through C—H⋯O inter­actions.

## Related literature

For the synthesis of 3-phenyl­rhodanine, see: Brown *et al.* (1956 ▶). For the therapeutic properties of rhodanine-based mol­ecules, including anti­convulsant, anti­bacterial, anti­viral and anti­diabetic properties, see: Momose *et al.* (1991 ▶); HCV protease, Sudo *et al.* (1997 ▶); HCV NS3 protease, Sing *et al.* (2001 ▶); aldols reductase, Bruno *et al.* (2002 ▶); factor protease, Sherida *et al.* (2006 ▶).
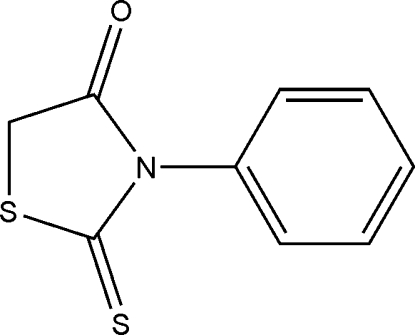


## Experimental

### 

#### Crystal data

C_9_H_7_NOS_2_
*M* *_r_* = 209.28
Monoclinic, 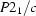

*a* = 12.9941 (13) Å
*b* = 5.6111 (6) Å
*c* = 12.7271 (13) Å
β = 93.847 (3)°
*V* = 925.86 (17) Å^3^
*Z* = 4
Mo *K*α radiation
μ = 0.53 mm^−1^
*T* = 296 (2) K
0.20 × 0.15 × 0.05 mm

#### Data collection

Bruker APEXII diffractometer
Absorption correction: multi-scan (*SADABS*; Bruker, 2000 ▶) *T*_min_ = 0.91, *T*_max_ = 0.97
10918 measured reflections
1800 independent reflections
1146 reflections with *I* > 2σ(*I*)
*R*_int_ = 0.066

#### Refinement

*R*[*F* ^2^ > 2σ(*F* ^2^)] = 0.040
*wR*(*F* ^2^) = 0.080
*S* = 1.00
1800 reflections
118 parameters
H-atom parameters constrained
Δρ_max_ = 0.25 e Å^−3^
Δρ_min_ = −0.23 e Å^−3^

### 

Data collection: *APEX2* (Bruker, 2004 ▶); cell refinement: *SAINT* (Bruker, 2004 ▶); data reduction: *SAINT*; program(s) used to solve structure: *SHELXS97* (Sheldrick, 2008 ▶); program(s) used to refine structure: *SHELXL97* (Sheldrick, 2008 ▶); molecular graphics: *ORTEP-3 for Windows* (Farrugia, 1997 ▶); software used to prepare material for publication: *SHELXL97* and *PLATON* (Spek, 2003 ▶).

## Supplementary Material

Crystal structure: contains datablocks global, I. DOI: 10.1107/S1600536808030079/pk2120sup1.cif

Structure factors: contains datablocks I. DOI: 10.1107/S1600536808030079/pk2120Isup2.hkl

Additional supplementary materials:  crystallographic information; 3D view; checkCIF report

## Figures and Tables

**Table 1:** Hydrogen-bond geometry (Å, °)

*D*—H⋯*A*	*D*—H	H⋯*A*	*D*⋯*A*	*D*—H⋯*A*
C5—H5⋯O1^i^	0.93	2.51	3.410 (3)	163
C8—H8⋯O1^ii^	0.93	2.46	3.386 (3)	171

Symmetry codes: (i) 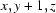; (ii) 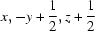.

**Fig. 1.**
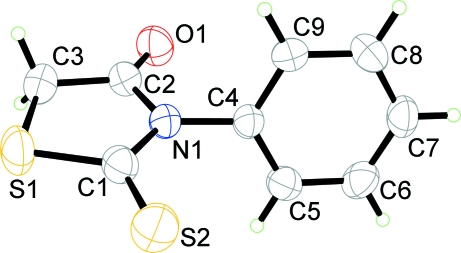
The asymmetric unit of 3-benzylrhodanine with atom labels and 50% probability displacement ellipsoids for non-H atoms.

## supplementary crystallographic information

### Comment

Rhodanine derivatives are attractive compounds owing to their outstanding biological activities. They have undergone rapid development as a result of their use in anticonvulsant, antibacterial, antiviral and antidiabetic treatments (Momose *et al.*, 1991). As an extension of these studies, we report herein on the structure of 3-phenylrhodanine (3-phenyl-2-thioxothiazolidin-4-one).

A 3-phenylrhodanine molecule, which is the asymmetric unit of the structure, is shown in Fig. 1. All the bond distances and bond angles are within the normal ranges.  The two parts of the molecule, the five-member heterocycle and the phenyl ring, are oriented at a dihedral angle of 72.3 (1)°. Adjacent molecules are connected through C–H—O hydrogen bonds (Table 1).

### Experimental

3-phenylrhodanine was synthesized according to the literature (Brown *et al.*, 1956), and was recrystallized using a mixed solvent of ether and 95% ethanol (1:1 by volume). Yellow sheet crystals are obtained.

### Refinement

All non-hydrogen atoms were found in Fourier maps, and were refined anisotropically. Hydrogen atoms were positioned geometrically, and the isotropic vibration parameters related to the atoms which they are bonded to with *U*_iso_ = 1.2 *U*_eq_.

### Crystal data

**Table:**

C_9_H_7_NOS_2_	*F*(000) = 432
*M**_r_* = 209.28	*D*_x_ = 1.501 Mg m^−^^3^
Monoclinic, *P*2_1_/*c*	Mo *K*α radiation, λ = 0.71073 Å
Hall symbol: -P 2ybc	Cell parameters from 1321 reflections
*a* = 12.9941 (13) Å	θ = 3.1–21.1°
*b* = 5.6111 (6) Å	µ = 0.53 mm^−^^1^
*c* = 12.7271 (13) Å	*T* = 296 K
β = 93.847 (3)°	Plate, yellow
*V* = 925.86 (17) Å^3^	0.20 × 0.15 × 0.05 mm
*Z* = 4	

### Data collection

**Table:**

Bruker APEXII diffractometer	1800 independent reflections
Radiation source: fine-focus sealed tube	1146 reflections with *I* > 2σ(*I*)
graphite	*R*_int_ = 0.066
Detector resolution: 8 pixels mm^-1^	θ_max_ = 26.0°, θ_min_ = 1.6°
ω scans	*h* = −14→15
Absorption correction: multi-scan (SADABS; Bruker, 2000)	*k* = −6→6
*T*_min_ = 0.91, *T*_max_ = 0.97	*l* = −15→15
10918 measured reflections	

### Refinement

**Table:**

Refinement on *F*^2^	Primary atom site location: structure-invariant direct methods
Least-squares matrix: full	Secondary atom site location: difference Fourier map
*R*[*F*^2^ > 2σ(*F*^2^)] = 0.040	Hydrogen site location: inferred from neighbouring sites
*wR*(*F*^2^) = 0.080	H-atom parameters constrained
*S* = 1.00	*w* = 1/[σ^2^(*F*_o_^2^) + (0.0272*P*)^2^ + 0.2182*P*] where *P* = (*F*_o_^2^ + 2*F*_c_^2^)/3
1800 reflections	(Δ/σ)_max_ < 0.001
118 parameters	Δρ_max_ = 0.25 e Å^−^^3^
0 restraints	Δρ_min_ = −0.23 e Å^−^^3^

### Special details

**Table:**

Geometry. All esds (except the esd in the dihedral angle between two l.s. planes) are estimated using the full covariance matrix. The cell esds are taken into account individually in the estimation of esds in distances, angles and torsion angles; correlations between esds in cell parameters are only used when they are defined by crystal symmetry. An approximate (isotropic) treatment of cell esds is used for estimating esds involving l.s. planes.
Refinement. Refinement of F^2^ against ALL reflections. The weighted R-factor wR and goodness of fit S are based on F^2^, conventional R-factors R are based on F, with F set to zero for negative F^2^. The threshold expression of F^2^ > 2sigma(F^2^) is used only for calculating R-factors(gt) etc. and is not relevant to the choice of reflections for refinement. R-factors based on F^2^ are statistically about twice as large as those based on F, and R- factors based on ALL data will be even larger.

### Fractional atomic coordinates and isotropic or equivalent isotropic displacement parameters (Å^2^)

**Table:**

	*x*	*y*	*z*	*U*_iso_*/*U*_eq_	
C1	0.35704 (19)	0.8242 (4)	0.64373 (18)	0.0412 (6)	
C2	0.2485 (2)	0.5060 (4)	0.58348 (17)	0.0413 (6)	
C3	0.32529 (19)	0.5020 (4)	0.50099 (19)	0.0495 (7)	
H3A	0.2908	0.5231	0.4317	0.059*	
H3B	0.3616	0.3508	0.5024	0.059*	
C4	0.20680 (17)	0.7118 (4)	0.74476 (17)	0.0351 (6)	
C5	0.13898 (18)	0.8984 (4)	0.74630 (18)	0.0433 (6)	
H5	0.1376	1.0140	0.6938	0.052*	
C6	0.07285 (19)	0.9134 (5)	0.8263 (2)	0.0478 (7)	
H6	0.0262	1.0389	0.8275	0.057*	
C7	0.0756 (2)	0.7437 (5)	0.90434 (19)	0.0484 (7)	
H7	0.0309	0.7549	0.9582	0.058*	
C8	0.1438 (2)	0.5586 (5)	0.90295 (19)	0.0512 (7)	
H8	0.1455	0.4441	0.9559	0.061*	
C9	0.21025 (19)	0.5414 (4)	0.82277 (18)	0.0444 (6)	
H9	0.2569	0.4158	0.8216	0.053*	
N1	0.27190 (14)	0.6844 (3)	0.65849 (14)	0.0364 (5)	
O1	0.17588 (14)	0.3754 (3)	0.58706 (13)	0.0541 (5)	
S1	0.41400 (5)	0.74161 (13)	0.52991 (6)	0.0566 (2)	
S2	0.40143 (5)	1.03944 (13)	0.71931 (6)	0.0583 (2)	

### Atomic displacement parameters (Å^2^)

**Table:**

	*U*^11^	*U*^22^	*U*^33^	*U*^12^	*U*^13^	*U*^23^
C1	0.0359 (15)	0.0436 (15)	0.0446 (14)	0.0026 (12)	0.0059 (11)	0.0053 (12)
C2	0.0456 (16)	0.0411 (15)	0.0378 (13)	0.0024 (13)	0.0066 (12)	0.0023 (12)
C3	0.0501 (16)	0.0522 (17)	0.0475 (14)	0.0041 (14)	0.0124 (12)	−0.0048 (13)
C4	0.0342 (14)	0.0356 (14)	0.0362 (12)	−0.0006 (11)	0.0077 (11)	−0.0010 (11)
C5	0.0440 (16)	0.0407 (15)	0.0458 (15)	0.0024 (13)	0.0069 (13)	0.0055 (11)
C6	0.0403 (16)	0.0461 (16)	0.0577 (16)	0.0089 (13)	0.0089 (13)	−0.0033 (13)
C7	0.0474 (16)	0.0539 (17)	0.0458 (14)	−0.0049 (15)	0.0182 (12)	−0.0063 (14)
C8	0.0614 (18)	0.0489 (16)	0.0445 (15)	−0.0046 (15)	0.0125 (14)	0.0097 (13)
C9	0.0497 (16)	0.0370 (14)	0.0472 (14)	0.0093 (12)	0.0090 (12)	0.0044 (12)
N1	0.0359 (12)	0.0356 (11)	0.0386 (11)	−0.0007 (9)	0.0088 (9)	−0.0003 (9)
O1	0.0594 (13)	0.0506 (11)	0.0532 (11)	−0.0147 (10)	0.0107 (9)	−0.0059 (9)
S1	0.0465 (4)	0.0673 (5)	0.0588 (4)	−0.0072 (4)	0.0228 (3)	−0.0061 (4)
S2	0.0511 (5)	0.0561 (5)	0.0682 (5)	−0.0128 (4)	0.0081 (4)	−0.0131 (4)

### Geometric parameters (Å, °)

**Table:**

C1—N1	1.379 (3)	C4—N1	1.439 (3)
C1—S2	1.626 (3)	C5—C6	1.378 (3)
C1—S1	1.733 (2)	C5—H5	0.9300
C2—O1	1.198 (3)	C6—C7	1.375 (3)
C2—N1	1.402 (3)	C6—H6	0.9300
C2—C3	1.496 (3)	C7—C8	1.366 (3)
C3—S1	1.793 (3)	C7—H7	0.9300
C3—H3A	0.9700	C8—C9	1.384 (3)
C3—H3B	0.9700	C8—H8	0.9300
C4—C5	1.370 (3)	C9—H9	0.9300
C4—C9	1.377 (3)		
			
N1—C1—S2	126.73 (18)	C6—C5—H5	120.3
N1—C1—S1	110.68 (17)	C7—C6—C5	120.3 (2)
S2—C1—S1	122.59 (15)	C7—C6—H6	119.9
O1—C2—N1	123.1 (2)	C5—C6—H6	119.9
O1—C2—C3	125.5 (2)	C8—C7—C6	120.2 (2)
N1—C2—C3	111.4 (2)	C8—C7—H7	119.9
C2—C3—S1	107.11 (17)	C6—C7—H7	119.9
C2—C3—H3A	110.3	C7—C8—C9	120.0 (2)
S1—C3—H3A	110.3	C7—C8—H8	120.0
C2—C3—H3B	110.3	C9—C8—H8	120.0
S1—C3—H3B	110.3	C4—C9—C8	119.5 (2)
H3A—C3—H3B	108.5	C4—C9—H9	120.3
C5—C4—C9	120.7 (2)	C8—C9—H9	120.3
C5—C4—N1	120.3 (2)	C1—N1—C2	116.9 (2)
C9—C4—N1	118.9 (2)	C1—N1—C4	124.14 (19)
C4—C5—C6	119.4 (2)	C2—N1—C4	119.0 (2)
C4—C5—H5	120.3	C1—S1—C3	93.86 (12)

### Hydrogen-bond geometry (Å, °)

**Table:**

*D*—H···*A*	*D*—H	H···*A*	*D*···*A*	*D*—H···*A*
C5—H5···O1^i^	0.93	2.51	3.410 (3)	163.
C8—H8···O1^ii^	0.93	2.46	3.386 (3)	171.

Symmetry codes: (i) *x*, *y*+1, *z*; (ii) *x*, −*y*+1/2, *z*+1/2.
